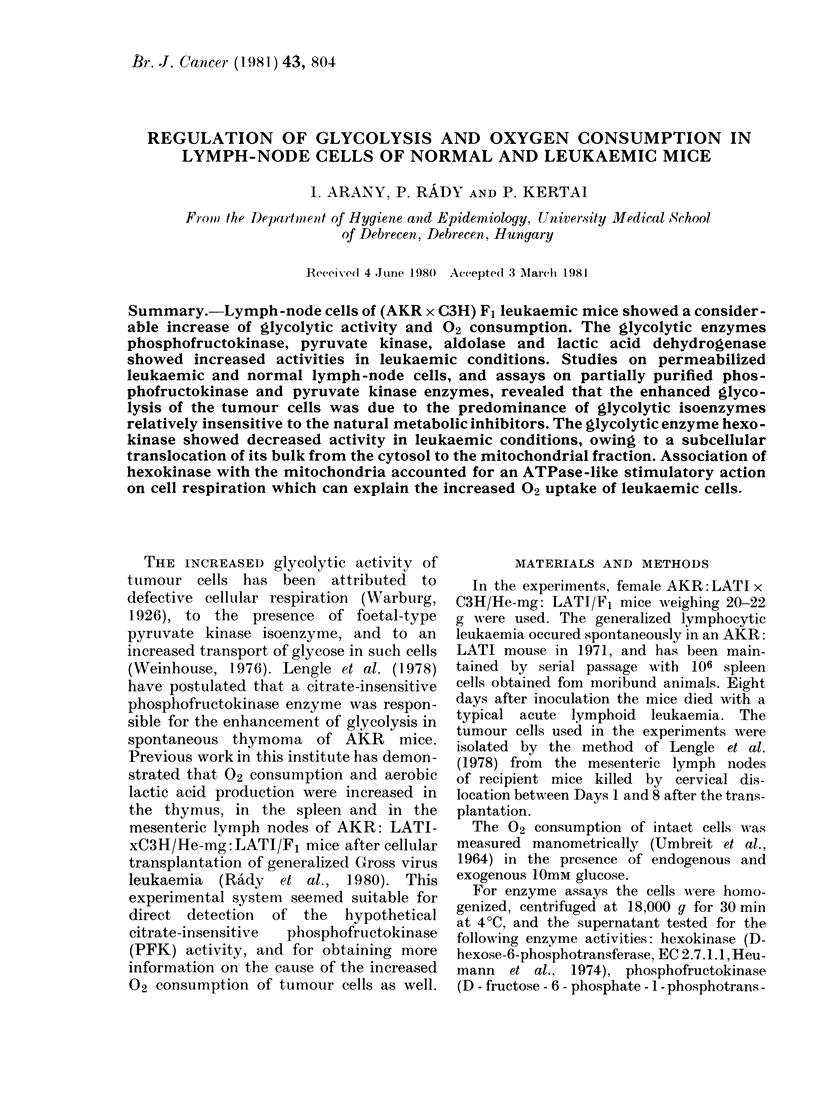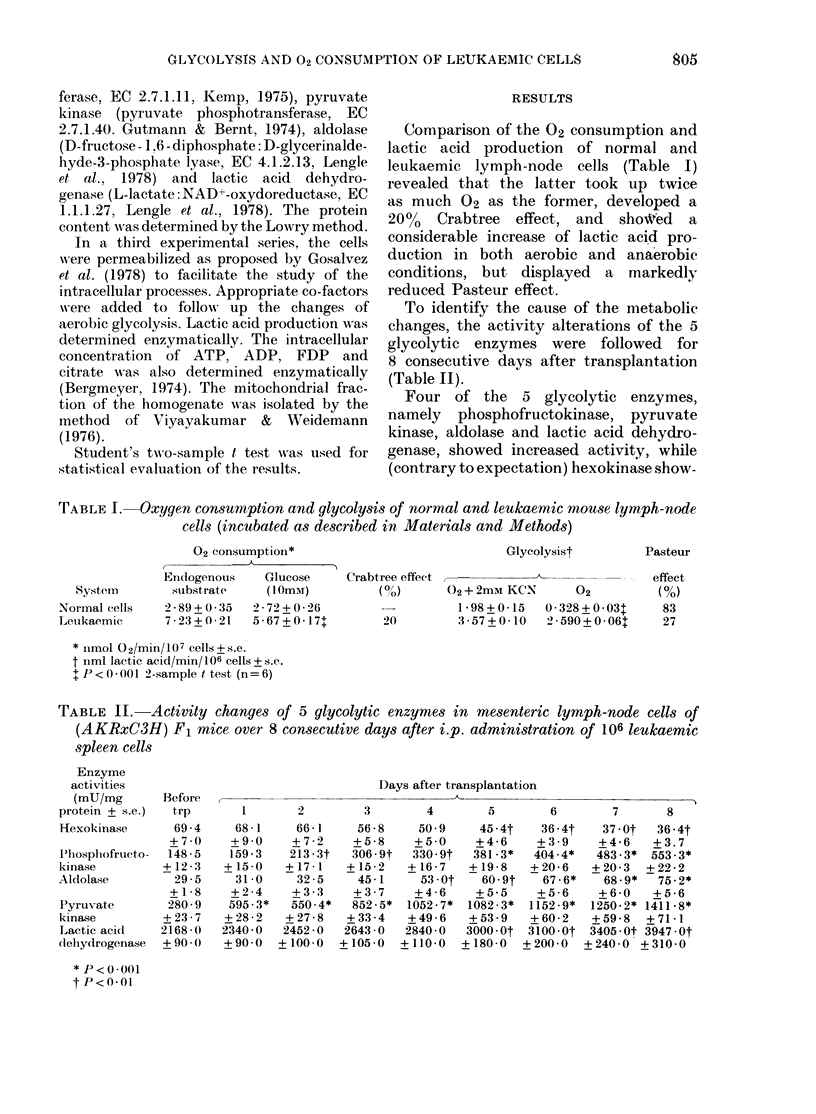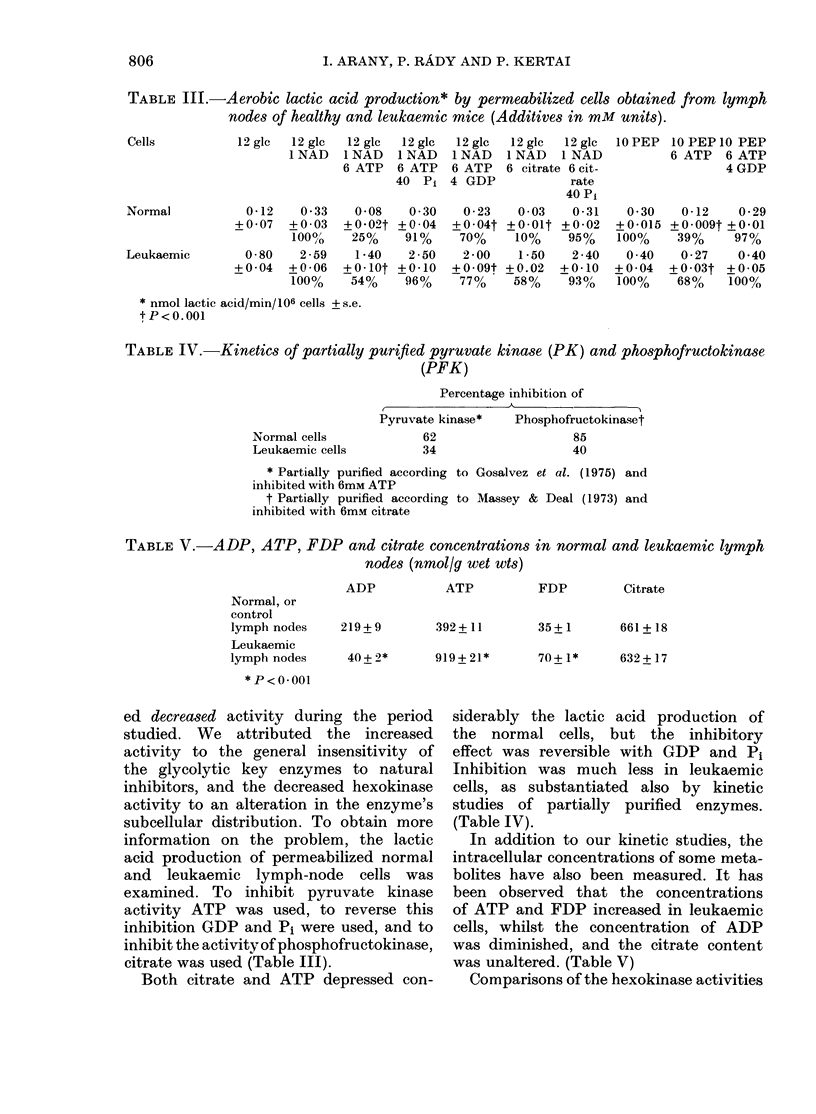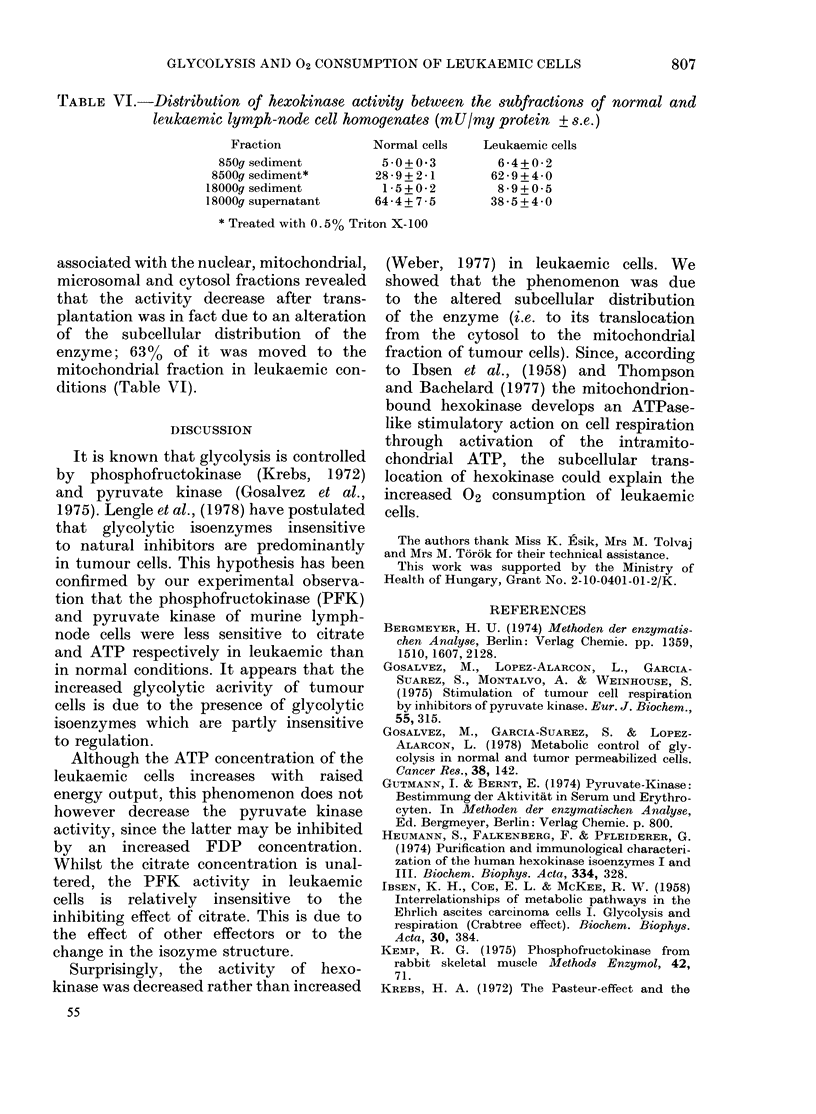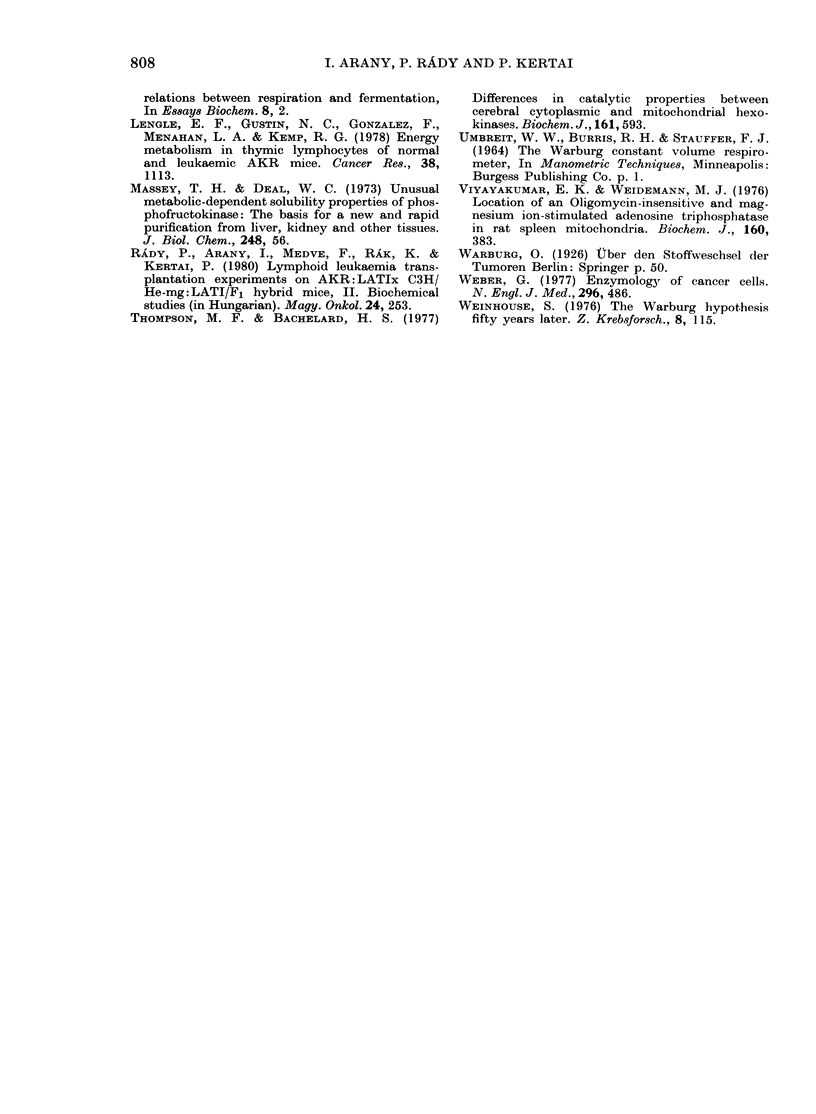# Regulation of glycolysis and oxygen consumption in lymph-node cells of normal and leukaemic mice.

**DOI:** 10.1038/bjc.1981.118

**Published:** 1981-06

**Authors:** I. Arany, P. Rády, P. Kertai

## Abstract

Lymph-node cells of (AKR X C3H) F1 leukaemic mice showed a considerable increase of glycolytic activity and O2 consumption. The glycolytic enzymes phosphofructokinase, pyruvate kinase, aldolase and lactic acid dehydrogenase showed increased activities in leukaemic conditions. Studies on permeabilized leukaemic and normal lymph-node cells, and assays on partially purified phosphofructokinase and pyruvate kinase enzymes, revealed that the enhanced glycolysis of the tumour cells was due to the predominance of glycolytic isoenzymes relatively insensitive to the natural metabolic inhibitors. The glycolytic enzyme hexokinase showed decreased activity in leukaemic conditions, owing to a subcellular translocation of its bulk from the cytosol to the mitochondrial fraction. Association of hexokinase with the mitochondria accounted for an ATPase-like stimulatory action on cell respiration which can explain the increased O2 uptake of leukaemic cells.


*Br. J. Cancer* (1981) **43**, 804

### REGULATION OF GLYCOLYSIS AND OXYGEN CONSUMPTION IN LYMPH-NODE CELLS OF NORMAL AND LEUKAEMIC MICE

I. ARANY, P. RÁDY AND P. KERTAI

*From the Department of Hygiene and Epidemiology, University Medical School of Debrecen, Debrecen, Hungary*

Received 4 June 1980 Accepted 3 March 1981

**Summary.—Lymph-node cells of (AKR × C3H) $F_1$ leukaemic mice showed a considerable increase of glycolytic activity and $O_2$ consumption. The glycolytic enzymes phosphofructokinase, pyruvate kinase, aldolase and lactic acid dehydrogenase showed increased activities in leukaemic conditions. Studies on permeabilized leukaemic and normal lymph-node cells, and assays on partially purified phosphofructokinase and pyruvate kinase enzymes, revealed that the enhanced glycolysis of the tumour cells was due to the predominance of glycolytic isoenzymes relatively insensitive to the natural metabolic inhibitors. The glycolytic enzyme hexokinase showed decreased activity in leukaemic conditions, owing to a subcellular translocation of its bulk from the cytosol to the mitochondrial fraction. Association of hexokinase with the mitochondria accounted for an ATPase-like stimulatory action on cell respiration which can explain the increased $O_2$ uptake of leukaemic cells.**

THE INCREASED glycolytic activity of tumour cells has been attributed to defective cellular respiration (Warburg, 1926), to the presence of foetal-type pyruvate kinase isoenzyme, and to an increased transport of glycose in such cells (Weinhouse, 1976). Lengle *et al.* (1978) have postulated that a citrate-insensitive phosphofructokinase enzyme was responsible for the enhancement of glycolysis in spontaneous thymoma of AKR mice. Previous work in this institute has demonstrated that $O_2$ consumption and aerobic lactic acid production were increased in the thymus, in the spleen and in the mesenteric lymph nodes of AKR: LATI-xC3H/He-mg:LATI/$F_1$ mice after cellular transplantation of generalized Gross virus leukaemia (Rády *et al.*, 1980). This experimental system seemed suitable for direct detection of the hypothetical citrate-insensitive phosphofructokinase (PFK) activity, and for obtaining more information on the cause of the increased $O_2$ consumption of tumour cells as well.

### MATERIALS AND METHODS

In the experiments, female AKR:LATI × C3H/He-mg: LATI/$F_1$ mice weighing 20–22 g were used. The generalized lymphocytic leukaemia occured spontaneously in an AKR: LATI mouse in 1971, and has been maintained by serial passage with $10^6$ spleen cells obtained fom moribund animals. Eight days after inoculation the mice died with a typical acute lymphoid leukaemia. The tumour cells used in the experiments were isolated by the method of Lengle *et al.* (1978) from the mesenteric lymph nodes of recipient mice killed by cervical dislocation between Days 1 and 8 after the transplantation.

The $O_2$ consumption of intact cells was measured manometrically (Umbreit *et al.*, 1964) in the presence of endogenous and exogenous 10mM glucose.

For enzyme assays the cells were homogenized, centrifuged at 18,000 *g* for 30 min at 4°C, and the supernatant tested for the following enzyme activities: hexokinase (D-hexose-6-phosphotransferase, EC 2.7.1.1, Heumann *et al.*, 1974), phosphofructokinase (D-fructose-6-phosphate-1-phosphotransferase, EC 2.7.1.11, Kemp, 1975), pyruvate kinase (pyruvate phosphotransferase, EC 2.7.1.40. Gutmann & Bernt, 1974), aldolase (D-fructose-1,6-diphosphate: D-glycerinaldehyde-3-phosphate lyase, EC 4.1.2.13, Lengle *et al.*, 1978) and lactic acid dehydrogenase (L-lactate: $NAD^+$-oxydoreductase, EC 1.1.1.27, Lengle *et al.*, 1978). The protein content was determined by the Lowry method.

In a third experimental series, the cells were permeabilized as proposed by Gosalvez *et al.* (1978) to facilitate the study of the intracellular processes. Appropriate co-factors were added to follow up the changes of aerobic glycolysis. Lactic acid production was determined enzymatically. The intracellular concentration of ATP, ADP, FDP and citrate was also determined enzymatically (Bergmeyer, 1974). The mitochondrial fraction of the homogenate was isolated by the method of Viyayakumar & Weidemann (1976).

Student's two-sample *t* test was used for statistical evaluation of the results.

### RESULTS

Comparison of the $O_2$ consumption and lactic acid production of normal and leukaemic lymph-node cells (Table I) revealed that the latter took up twice as much $O_2$ as the former, developed a 20% Crabtree effect, and showed a considerable increase of lactic acid production in both aerobic and anaerobic conditions, but displayed a markedly reduced Pasteur effect.

To identify the cause of the metabolic changes, the activity alterations of the 5 glycolytic enzymes were followed for 8 consecutive days after transplantation (Table II).

Four of the 5 glycolytic enzymes, namely phosphofructokinase, pyruvate kinase, aldolase and lactic acid dehydrogenase, showed increased activity, while (contrary to expectation) hexokinase show-

TABLE I.—*Oxygen consumption and glycolysis of normal and leukaemic mouse lymph-node cells (incubated as described in Materials and Methods)*

| | $O_2$ consumption* | | | Glycolysis† | | Pasteur |
|---|---|---|---|---|---|---|
| System | Endogenous substrate | Glucose (10mM) | Crabtree effect (%) | $O_2$ + 2mM KCN | $O_2$ | effect (%) |
| Normal cells | 2·89 ± 0·35 | 2·72 ± 0·26 | — | 1·98 ± 0·15 | 0·328 ± 0·03‡ | 83 |
| Leukaemic | 7·23 ± 0·21 | 5·67 ± 0·17‡ | 20 | 3·57 ± 0·10 | 2·590 ± 0·06‡ | 27 |

* nmol $O_2$/min/$10^7$ cells ± s.e.

† nml lactic acid/min/$10^6$ cells ± s.e.

‡ $P < 0·001$ 2-sample $t$ test (n = 6)

TABLE II.—*Activity changes of 5 glycolytic enzymes in mesenteric lymph-node cells of (AKRxC3H) $F_1$ mice over 8 consecutive days after i.p. administration of $10^6$ leukaemic spleen cells*

| Enzyme activities (mU/mg protein ± s.e.) | Before trp | Days after transplantation 1 | 2 | 3 | 4 | 5 | 6 | 7 | 8 |
|---|---|---|---|---|---|---|---|---|---|
| Hexokinase | 69·4 | 68·1 | 66·1 | 56·8 | 50·9 | 45·4† | 36·4† | 37·0† | 36·4† |
| | ± 7·0 | ± 9·0 | ± 7·2 | ± 5·8 | ± 5·0 | ± 4·6 | ± 3·9 | ± 4·6 | ± 3.7 |
| Phosphofructo- | 148·5 | 159·3 | 213·3† | 306·9† | 330·9† | 381·3* | 404·4* | 483·3* | 553·3* |
| kinase | ± 12·3 | ± 15·0 | ± 17·1 | ± 15·2 | ± 16·7 | ± 19·8 | ± 20·6 | ± 20·3 | ± 22·2 |
| Aldolase | 29·5 | 31·0 | 32·5 | 45·1 | 53·0† | 60·9† | 67·6* | 68·9* | 75·2* |
| | ± 1·8 | ± 2·4 | ± 3·3 | ± 3·7 | ± 4·6 | ± 5·5 | ± 5·6 | ± 6·0 | ± 5·6 |
| Pyruvate | 280·9 | 595·3* | 550·4* | 852·5* | 1052·7* | 1082·3* | 1152·9* | 1250·2* | 1411·8* |
| kinase | ± 23·7 | ± 28·2 | ± 27·8 | ± 33·4 | ± 49·6 | ± 53·9 | ± 60·2 | ± 59·8 | ± 71·1 |
| Lactic acid | 2168·0 | 2340·0 | 2452·0 | 2643·0 | 2840·0 | 3000·0† | 3100·0† | 3405·0† | 3947·0† |
| dehydrogenase | ± 90·0 | ± 90·0 | ± 100·0 | ± 105·0 | ± 110·0 | ± 180·0 | ± 200·0 | ± 240·0 | ± 310·0 |

* $P < 0·001$
† $P < 0·01$

TABLE III.—*Aerobic lactic acid production* by permeabilized cells obtained from lymph nodes of healthy and leukaemic mice (Additives in mM units).*

| Cells | 12 glc | 12 glc 1 NAD | 12 glc 1 NAD 6 ATP | 12 glc 1 NAD 6 ATP 40 $P_i$ | 12 glc 1 NAD 6 ATP 4 GDP | 12 glc 1 NAD 6 citrate | 12 glc 1 NAD 6 citrate 40 $P_i$ | 10 PEP | 10 PEP 6 ATP | 10 PEP 6 ATP 4 GDP |
|---|---|---|---|---|---|---|---|---|---|---|
| Normal | 0·12 ±0·07 | 0·33 ±0·03 100% | 0·08 ±0·02† 25% | 0·30 ±0·04 91% | 0·23 ±0·04† 70% | 0·03 ±0·01† 10% | 0·31 ±0·02 95% | 0·30 ±0·015 100% | 0·12 ±0·009† 39% | 0·29 ±0·01 97% |
| Leukaemic | 0·80 ±0·04 | 2·59 ±0·06 100% | 1·40 ±0·10† 54% | 2·50 ±0·10 96% | 2·00 ±0·09† 77% | 1·50 ±0.02 58% | 2·40 ±0·10 93% | 0·40 ±0·04 100% | 0·27 ±0·03† 68% | 0·40 ±0·05 100% |

* nmol lactic acid/min/$10^6$ cells ±s.e.
† $P < 0.001$

TABLE IV.—*Kinetics of partially purified pyruvate kinase (PK) and phosphofructokinase (PFK)*

| | Percentage inhibition of | |
|---|---|---|
| | Pyruvate kinase* | Phosphofructokinase† |
| Normal cells | 62 | 85 |
| Leukaemic cells | 34 | 40 |

* Partially purified according to Gosalvez *et al.* (1975) and inhibited with 6mM ATP

† Partially purified according to Massey & Deal (1973) and inhibited with 6mM citrate

TABLE V.—*ADP, ATP, FDP and citrate concentrations in normal and leukaemic lymph nodes (nmol/g wet wts)*

| | ADP | ATP | FDP | Citrate |
|---|---|---|---|---|
| Normal, or control lymph nodes | 219 ± 9 | 392 ± 11 | 35 ± 1 | 661 ± 18 |
| Leukaemic lymph nodes | 40 ± 2* | 919 ± 21* | 70 ± 1* | 632 ± 17 |

* $P < 0·001$

ed *decreased* activity during the period studied. We attributed the increased activity to the general insensitivity of the glycolytic key enzymes to natural inhibitors, and the decreased hexokinase activity to an alteration in the enzyme's subcellular distribution. To obtain more information on the problem, the lactic acid production of permeabilized normal and leukaemic lymph-node cells was examined. To inhibit pyruvate kinase activity ATP was used, to reverse this inhibition GDP and $P_i$ were used, and to inhibit the activity of phosphofructokinase, citrate was used (Table III).

Both citrate and ATP depressed considerably the lactic acid production of the normal cells, but the inhibitory effect was reversible with GDP and $P_i$ Inhibition was much less in leukaemic cells, as substantiated also by kinetic studies of partially purified enzymes. (Table IV).

In addition to our kinetic studies, the intracellular concentrations of some metabolites have also been measured. It has been observed that the concentrations of ATP and FDP increased in leukaemic cells, whilst the concentration of ADP was diminished, and the citrate content was unaltered. (Table V)

Comparisons of the hexokinase activities

TABLE VI.—*Distribution of hexokinase activity between the subfractions of normal and leukaemic lymph-node cell homogenates (mU/my protein ± s.e.)*

| Fraction | Normal cells | Leukaemic cells |
|---|---|---|
| 850*g* sediment | 5·0±0·3 | 6·4±0·2 |
| 8500*g* sediment* | 28·9±2·1 | 62·9±4·0 |
| 18000*g* sediment | 1·5±0·2 | 8·9±0·5 |
| 18000*g* supernatant | 64·4±7·5 | 38·5±4·0 |

* Treated with 0.5% Triton X-100

associated with the nuclear, mitochondrial, microsomal and cytosol fractions revealed that the activity decrease after transplantation was in fact due to an alteration of the subcellular distribution of the enzyme; 63% of it was moved to the mitochondrial fraction in leukaemic conditions (Table VI).

### DISCUSSION

It is known that glycolysis is controlled by phosphofructokinase (Krebs, 1972) and pyruvate kinase (Gosalvez *et al.*, 1975). Lengle *et al.*, (1978) have postulated that glycolytic isoenzymes insensitive to natural inhibitors are predominantly in tumour cells. This hypothesis has been confirmed by our experimental observation that the phosphofructokinase (PFK) and pyruvate kinase of murine lymph-node cells were less sensitive to citrate and ATP respectively in leukaemic than in normal conditions. It appears that the increased glycolytic acrivity of tumour cells is due to the presence of glycolytic isoenzymes which are partly insensitive to regulation.

Although the ATP concentration of the leukaemic cells increases with raised energy output, this phenomenon does not however decrease the pyruvate kinase activity, since the latter may be inhibited by an increased FDP concentration. Whilst the citrate concentration is unaltered, the PFK activity in leukaemic cells is relatively insensitive to the inhibiting effect of citrate. This is due to the effect of other effectors or to the change in the isozyme structure.

Surprisingly, the activity of hexokinase was decreased rather than increased (Weber, 1977) in leukaemic cells. We showed that the phenomenon was due to the altered subcellular distribution of the enzyme (*i.e.* to its translocation from the cytosol to the mitochondrial fraction of tumour cells). Since, according to Ibsen *et al.*, (1958) and Thompson and Bachelard (1977) the mitochondrion-bound hexokinase develops an ATPase-like stimulatory action on cell respiration through activation of the intramitochondrial ATP, the subcellular translocation of hexokinase could explain the increased $O_2$ consumption of leukaemic cells.

The authors thank Miss K. Ésik, Mrs M. Tolvaj and Mrs M. Török for their technical assistance.

This work was supported by the Ministry of Health of Hungary, Grant No. 2-10-0401-01-2/K.

### REFERENCES

BERGMEYER, H. U. (1974) *Methoden der enzymatischen Analyse*, Berlin: Verlag Chemie. pp. 1359, 1510, 1607, 2128.

GOSALVEZ, M., LOPEZ-ALARCON, L., GARCIA-SUAREZ, S., MONTALVO, A. & WEINHOUSE, S. (1975) Stimulation of tumour cell respiration by inhibitors of pyruvate kinase. *Eur. J. Biochem.*, **55**, 315.

GOSALVEZ, M., GARCIA-SUAREZ, S. & LOPEZ-ALARCON, L. (1978) Metabolic control of glycolysis in normal and tumor permeabilized cells. *Cancer Res.*, **38**, 142.

GUTMANN, I. & BERNT, E. (1974) Pyruvate-Kinase: Bestimmung der Aktivität in Serum und Erythrocyten. In *Methoden der enzymatischen Analyse*, Ed. Bergmeyer, Berlin: Verlag Chemie. p. 800.

HEUMANN, S., FALKENBERG, F. & PFLEIDERER, G. (1974) Purification and immunological characterization of the human hexokinase isoenzymes I and III. *Biochem. Biophys. Acta*, **334**, 328.

IBSEN, K. H., COE, E. L. & MCKEE, R. W. (1958) Interrelationships of metabolic pathways in the Ehrlich ascites carcinoma cells I. Glycolysis and respiration (Crabtree effect). *Biochem. Biophys. Acta*, **30**, 384.

KEMP, R. G. (1975) Phosphofructokinase from rabbit skeletal muscle *Methods Enzymol*, **42**, 71.

KREBS, H. A. (1972) The Pasteur-effect and the relations between respiration and fermentation, In *Essays Biochem.* **8**, 2.

LENGLE, E. F., GUSTIN, N. C., GONZALEZ, F., MENAHAN, L. A. & KEMP, R. G. (1978) Energy metabolism in thymic lymphocytes of normal and leukaemic AKR mice. *Cancer Res.*, **38**, 1113.

MASSEY, T. H. & DEAL, W. C. (1973) Unusual metabolic-dependent solubility properties of phosphofructokinase: The basis for a new and rapid purification from liver, kidney and other tissues. *J. Biol. Chem.*, **248**, 56.

RÁDY, P., ARANY, I., MEDVE, F., RÁK, K. & KERTAI, P. (1980) Lymphoid leukaemia transplantation experiments on AKR:LATIx C3H/He-mg:LATI/$F_1$ hybrid mice, II. Biochemical studies (in Hungarian). *Magy. Onkol.* **24**, 253.

THOMPSON, M. F. & BACHELARD, H. S. (1977) Differences in catalytic properties between cerebral cytoplasmic and mitochondrial hexokinases. *Biochem. J.*, **161**, 593.

UMBREIT, W. W., BURRIS, R. H. & STAUFFER, F. J. (1964) The Warburg constant volume respirometer, In *Manometric Techniques*, Minneapolis: Burgess Publishing Co. p. 1.

VIYAYAKUMAR, E. K. & WEIDEMANN, M. J. (1976) Location of an Oligomycin-insensitive and magnesium ion-stimulated adenosine triphosphatase in rat spleen mitochondria. *Biochem. J.*, **160**, 383.

WARBURG, O. (1926) Über den Stoffweschsel der Tumoren Berlin: Springer p. 50.

WEBER, G. (1977) Enzymology of cancer cells. *N. Engl. J. Med.*, **296**, 486.

WEINHOUSE, S. (1976) The Warburg hypothesis fifty years later. *Z. Krebsforsch.*, **8**, 115.